# Effect of bone cement sealing of the intramedullary femoral canal on coagulation status after total knee arthroplasty: a retrospective thromboelastography study

**DOI:** 10.1186/s13018-023-03942-y

**Published:** 2023-07-31

**Authors:** Jiahao Chen, Qiang Zhang, Hu Wang, Yingjin Sun, Ning Liu, Xiang-Yang Chen, Shuai Zhao, Shuo Feng

**Affiliations:** Department of Orthopedic Surgery, Affiliated Hospital of Xuzhou Medical University, 99 Huaihai Road, Xuzhou, 221002 Jiangsu China

**Keywords:** Total knee arthroplasty, Thromboelastography, Coagulation status, Bleeding

## Abstract

**Background:**

The main objective of this study was to investigate whether the use of bone cement in total knee arthroplasty (TKA) has an effect on postoperative coagulation status and bleeding.

**Methods:**

153 patients who underwent unilateral TKA between September 2019 and February 2023 were collected and divided into Bone and Cement&Bone groups according to whether bone cement was used to seal the bone medullary canal intraoperatively. Routine blood and thromboelastography (TEG) examinations were performed on the day before, the first day and the seventh day after surgery; postoperative bleeding, drainage, transfusion rate and the number of people suffering from deep venous thrombosis (DVT) were recorded.

**Results:**

There were no significant differences between the two groups in terms of baseline clinical characteristics before surgery (*P* > 0.05). In terms of TEG indicators, the coagulation index (CI) of the Bone&Cement group was lower than that of the Bone group on the first postoperative day and on the seventh postoperative day (*P* < 0.05). The CI of patients in the Bone group on the first postoperative day was lower than that of the preoperative day (*P* < 0.05); in terms of blood loss, the total blood loss and occult blood loss were lower in the Bone&Cement group than in the Bone group (*P* < 0.05). In addition, there was no significant difference in postoperative drainage,transfusion rate and the incidence of DVT between the two groups.

**Conclusion:**

Blocking the intramedullary canal of the femur with bone cement during TKA improves relative postoperative hypocoagulation and reduces postoperative blood loss, although there is no significant effect on transfusion rates, drainage and DVT.

## Introduction

Total knee arthroplasty (TKA) is a reliable and effective treatment for end-stage osteoarthritis of the knee [1], providing significant relief from knee pain and improving function. Haemorrhagic anaemia is a common complication after TKA [2]. Bleeding from the medullary cavity after expansion is a very important cause of blood loss after knee arthroplasty。In total knee arthroplasty the femoral medullary cavity  needs to be opened, and an open femoral medullary cavity can cause blood loss. Previous literature has shown that there are different ways of dealing with an open femoral medullary cavity  (no treatment, sealing with bone or cement) and that these ways have different effects on postoperative blood loss. Therefore, the management of the open femoral medullary cavity  is important to reduce postoperative bleeding. In recent years clinical methods have been used to reduce bleeding by sealing the femoral marrow cavity. Batmaz et al. found that sealing the intramedullary canal with autologous bone graft reduced postoperative blood loss [3]. İsmail Dikmen found the same effect with bone cement, but in his study he found that sealing the canal with autologous bone had no effect on blood loss [4], while other authors have concluded that none of these methods reduced postoperative blood loss [5] in addition to the conflicting results on postoperative drainage and transfusion rates in related articles [4–7].

Thromboelastography (TEG) is a novel method used to detect blood viscosity in vitro [8]. The principle is to simulate the whole process of whole blood in vivo from clot formation to dissolution, which is plotted as a dynamic curve image by an image sensing system. It is widely used as a tool to evaluate blood coagulation status, blood composition and antithrombotic drugs, providing information on coagulation, fibrinolysis and platelet count and function, thus assessing the clinical risk of thrombosis or bleeding and guiding patients to individualised treatment with anticoagulation, bleeding and fibrinolytic drugs. In recent years, TEG has been increasingly used in the perioperative assessment of coagulation status in total knee arthroplasty [9–13].

Previous studies have demonstrated that either bone or cement sealing of the medullary canal improves postoperative blood loss compared to leaving the canal empty, but there is debate as to whether there is a difference in the effect of the two on postoperative blood loss, transfusion rates and coagulation status of patients. This study therefore retrospectively analyzed the case data of patients who had their femoral canal sealed with an autologous osteotomy block and an autologous osteotomy block combined with bone cement during TKA. TEG was used to analyze the differences in coagulation status between the two groups and to compare the total blood loss, hidden blood loss, allograft transfusion rate and incidence of deep venous thrombosis (DVT) between the two groups.

## Materials and methods

### Patients and groups

This was a retrospective case–control study and was approved by the Ethics Committee of the Affiliated Hospital of Xuzhou Medical University. 155 patients who underwent unilateral primary total knee arthroplasty between September 2019 and February 2023 were collected for study analysis. All patients included were clearly diagnosed with unilateral knee osteoarthritis and had failed conservative treatment, those with no previous history of knee surgery, and those with comorbid but well-controlled medical systemic disease. Patients who had received anticoagulant medication within half a month, had a bleeding tendency, or had incomplete medical records were excluded, as were patients with co-morbidities such as rheumatoid arthritis, cancer, or haematological disorders affecting the function of the coagulation system. In one group of patients, the bone canal was closed with a bone plug, made from an autogenous osteotomy block with a cross-section slightly larger than the intramedullary locus of the femur so that it could be completely filled, and then the femoral canal was sealed with bone cement. In this experiment, 80 patients (femoral bone medullary cavity was sealed by bone and cement) and 75 patients (femoral bone medullary cavity was sealed by bone) were divided into two groups. During the postoperative follow-up, two patients in the group (75) were diagnosed with rheumatoid arthritis, which has an effect on coagulation, and therefore were excluded.

### Surgical procedure and peri‑operative management

All surgeries were performed by the same surgical team under a tourniquet. Patients were anaesthetised generally with a medial parapatellar approach. In one group of patients, the femoral medullary canal was sealed with a bone plug made from an autologous osteotomy, while in the other group, the femoral medullary canal was sealed with bone cement on top of a bone plug made from an autologous osteotomy. The prosthesis was coated with bone cement, the prosthesis was placed, the canal was drained, and the incision was closed layer by layer with a cotton pad and pressure dressing. The drainage tube was completely clamped for 2 h after the operation, and the drainage flow was regularly observed and the degree of drainage tube closure was adjusted according to the amount of drainage flow. All patients with TKA were given oral rivaroxaban 10 mg 6 h after surgery, once daily until discharge. After discharge, in the absence of bleeding events, rivaroxaban 10 mg/d was given for 4 weeks to prevent DVT and was discontinued in patients who developed postoperative ecchymosis. Erythropoietin was not used to improve anaemia during hospitalisation. All patients had a venous ultrasound of the lower limbs performed by the same experienced ultrasonographer on the seventh postoperative day, and those with venous thrombosis of the lower limbs were promptly referred to the vascular surgery department for appropriate management.

### Parameters

To the best of our knowledge, we were the first to introduce TEG to the study of postoperative coagulation status in TKA patients with different ways of blocking the femoral canal. Among the parameters of the TEG, reaction time(R) represents the time required for the body to initiate the clotting pathway to fibrin production; clotting time(K) value represents the time required for fibrin processing and modification into a clot; the alpha-angle is a measure of the rate of clot formation; the maximal amplitude of the trace(MA) shows the maximum strength of the clot produced; and the coagulation index(CI) reflects the general picture of the whole clotting process, which is calculated as CI = − 0.6516R − 0.3772 K + 0.1224MA + 0.0759Angle − 7.7922. Patients underwent TEG and complete blood count preoperatively and on the first day and seven days postoperatively. Intraoperative bleeding and total drainage after extubation were recorded and the number of allogeneic transfusions in the perioperative period was recorded. Total blood loss by day seven postoperatively calculated according to Gross [14] and Nadler's formula in (Table 3). Hidden blood loss is equal to the calculated total blood loss minus the visible blood loss, and if the patient has a post-operative transfusion, then the volume of the transfusion is also counted [15]. Record the number of patients transfused, the amount of fluid drained and compare the incidence of DVT and wound complications between the two groups during hospitalization.

### Indications for allogeneic blood transfusion

The protocols of the perioperative blood transfusion guidelines provided by the Chinese Ministry of Health are used as criteria: 1. the patient's haemoglobin (HB) concentration is < 70 g/L; 2. when the patient develops any symptoms associated with anaemia such as dizziness and weakness, palpitations and breathlessness. The attending physician needs to make an assessment of the need for blood transfusion for each patient.

### Statistical analysis

SPSS 25.0 software (IBM Corporation) was used for statistical analysis in this study. Categorical variables were expressed as absolute numbers and percentages, the Kolmogorov-Smirnoff test was used to determine whether the data were normally distributed, and continuous variables were expressed as mean ± standard deviation. Continuous variables were compared between the two groups using the independent samples t-test, and comparisons of TEG indicators between patients in the same group at different times were expressed using GraphPad Prism 8.01 bar charts. A chi-square test was used to compare transfusion rates and DVT incidence between groups. *P*-values < 0.05 were considered statistically significant.

## Results

### Patients’ characteristics

We selected 153 patients, 53 men and 100 women, from those treated with unilateral TKA from September 2019 to February 2023 for inclusion in the study. At postoperative follow-up, patients who developed DVT resolved with anticoagulation, and no complications such as prosthetic joint infection and prosthetic loosening occurred at follow-up.

There were no significant differences between the two groups in terms of age, gender, BMI, time to surgery and preoperative Hb and homozygous cell typing (HCT) (Table1).

**Table 1:** Baseline characteristics of patients

	Bone&Cement group	Bone group	*P* value
n	80	73	
Age (years)	62.04 ± 6.93	63.63 ± 6.36	0.145
Sex			0.109
Male	23	30	
Female	57	43	
BMI (kg/m^2^)	27.89 ± 3.49	28.64 ± 3.38	0.182
Surgical time (min)	68.32 ± 6.06	69.95 ± 6.10	0.099
Hb (g/L)	129.04 ± 7.88	128.87 ± 8.69	0.899
HCT	38.38 ± 2.81	37.79 ± 3.00	0.213

The gender variable was calculated using the chi-square test and the remaining variables were calculated using the independent samples t-test

^*^P < 0.05

*BMI* body mass index

### TEG characteristics of patients with two group

**Fig. 1:**
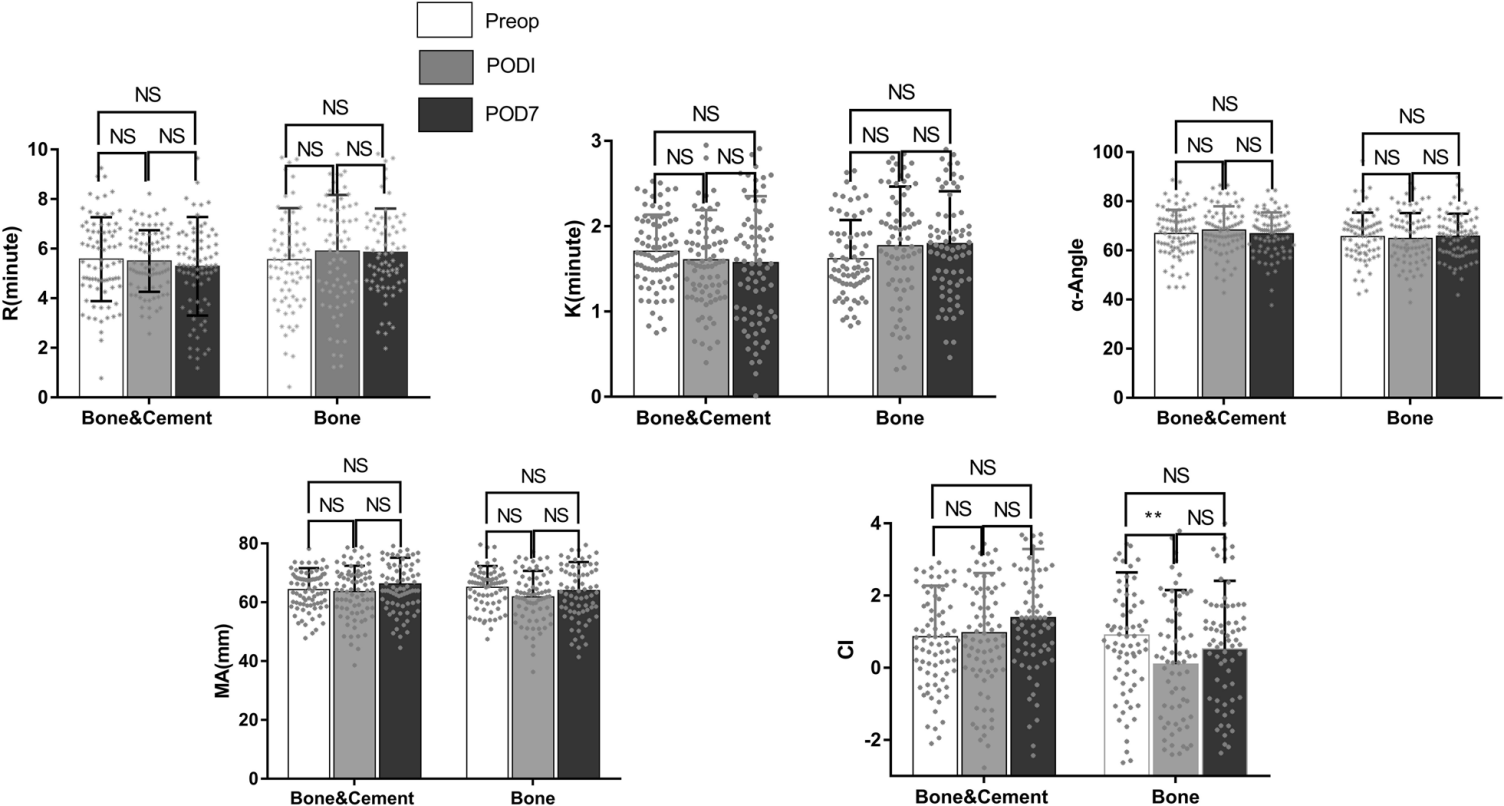
Comparison of different times regarding TEG values between two groups. Data are mean ± SD, *P*-values were calculated by one-way ANOVA, *P < 0.05, **P < 0.01, ****P < 0.0001, NS: No significance

No significant differences were found in the preoperative TEG indicators between the two groups. α-angle was higher in the Bone group than in the Bone&Cement group on the first postoperative day; the CI was lower in the Bone&Cement group than in the Bone group on the first postoperative day and on the seventh postoperative day, and the differences were statistically significant (Table 2), and the CI was lower in the Bone group on the first postoperative day than before surgery (Fig. 1). The difference was statistically significant, indicating that patients in the Bone group had a relatively lower coagulation status postoperatively than those in the Bone&Cement group (Table 3).

**Table 2:** TEG valuables at different time points

	Time points	Bone&Cement group	Bone group	*P* value
R (min)	Pre-op	5.58 ± 1.69	5.56 ± 2.08	0.938
	POD1	5.50 ± 1.24	5.85 ± 2.40	0.254
	POD7	5.23 ± 2.13	5.85 ± 1.77	0.052
K (min)	Pre-op	1.71 ± 0.43	1.62 ± 0.45	0.226
	POD1	1.61 ± 0.58	1.77 ± 0.70	0.112
	POD7	1.57 ± 0.78	1.79 ± 0.62	0.055
α-angle (º)	Pre-op	66.90 ± 9.60	65.63 ± 9.74	0.416
	POD1	68.33 ± 9.66	64.91 ± 10.38	0.036*
	POD7	66.82 ± 8.63	65.80 ± 9.20	0.479
MA (mm)	Pre-op	64.31 ± 7.30	65.07 ± 7.29	0.521
	POD1	63.66 ± 8.79	61.85 ± 8.85	0.207
	POD7	66.21 ± 8.95	63.98 ± 9.69	0.141
CI	Pre-op	0.86 ± 1.42	0.90 ± 1.74	0.862
	POD1	0.97 ± 1.66	0.20 ± 2.05	0.012*
	POD7	1.39 ± 1.90	0.52 ± 1.89	0.005*

*P < 0.05; R, reaction time; K, clotting time; MA, maximum amplitude; CI, coagulation index; POD1, first postoperative day; POD7, seventh postoperative day; the TEG indicators were calculated using the independent samples t-test

**Table 3:** Gross’s and Nadler’s formula

Methods	Formula	
Gross’s formula	TBL = EBV(HCT_preop_ − HCT_postop_)/HCT_ave_	TBL = calculated total blood loss (mL)
		HCT_ave_ = average of the pre-operative and the lowest post-operative Hct levels
Nadler’s formula	EBV = 0.3669H^3^ + 0.03219W + 0.6041* = 0.3561H^3^ + 0.03308W + 0.1833**	EBV = estimated blood volume(mL)
		H = height(m)
		W = weight(kg)

*For male

**For female

### Blood loss and post‑operative complications

The postoperative transfusion rate and the incidence of DVT were not significantly different between the two groups. In contrast, the total blood loss and occult blood loss in the Bone&Cement group was lower than that in the Bone group, and the difference was statistically significant. The mean hidden blood loss in the Bone&Cement group was 739.96, standing at 59.74% of the total blood loss, while the Bone group accounted for approximately 64.54%, which further corroborated the Bone group's relatively low coagulation status in the postoperative period. There was also no significant difference in intraoperative bleeding and drainage (Table4).

**Table 4:** Postoperative blood loss indicators, transfusion rate and DVT results

	Bone&Cement group	Bone group	*P* value
Calculated blood loss (mL)	1238.64 ± 308.51	1353.03 ± 339.34	0.030*
Intraoperative blood loss (mL)	120.73 ± 59.38	118.46 ± 42.52	0.788
Drain blood volume (mL)	377.95 ± 87.52	361.35 ± 83.60	0.233
Hidden blood loss (mL)	739.96 ± 327.96	873.21 ± 350.36	0.016*
Transfusion rate	7 (8.75%)	11 (15.07%)	0.226
DVT	2 (2.5%)	1 (1.37%)	0.615

Transfusion rate and DVT were calculated using the chi-square test and the remaining variables were calculated using the independent samples t-test

*DVT* deep venous thrombosis

*P < 0.05

## Discussion

In our study we evaluated the effect of two different ways of sealing the femoral canal on patients' postoperative coagulation status and blood loss. By comparing TEG indicators (preoperative, first postoperative day and seventh postoperative day), postoperative blood loss, transfusion rate and DVT incidence, we found that patients without bone cement sealing of the canal had a relatively hypocoagulable postoperative state and had more total and hidden blood loss in the postoperative period than the other group. These results were statistically significant. In addition, bone cement did not alter postoperative drainage, transfusion rates or the incidence of DVT.

The use of TEG for monitoring post-operative coagulation status in orthopaedic arthroplasty has become increasingly widespread in recent years [16, 17]. Compared to traditional coagulation tests, TEG focuses on the function of blood components and allows for a full picture of coagulation, providing a dynamic, comprehensive view of the entire coagulation response and providing better insight than conventional coagulation tests in assessing a patient's coagulation status [9, 18, 19]. Studies show that TEG is very reliable in monitoring coagulation status after orthopaedic surgery [17]. In a study by Joshua Gary [20] of 1818 orthopaedic trauma patients who underwent thromboelastography, they found that MA was an independent predictor of thromboembolism in patients. Brill et al. [21] found that patients who were eligible for hypercoagulable TEG had a nearly doubled incidence of DVT. In a thromboelastography study of post-TKA ecchymosis by Wang et al. [22], changes in CI values were found to be an independent risk factor for the development of ecchymosis. In our study there was a significant difference between the two groups in terms of postoperative CI, which in turn reflected the overall coagulation status, with the Bone Group and Bone&Cement group comparing relatively low coagulation, suggesting that the use of bone cement had an effect on the coagulation status of the patients in the postoperative period, which was further illustrated by the comparison of total and occult blood loss in the two groups in the postoperative period.

The blood loss in the perioperative period of TKA is large, and it has been reported that the blood loss after TKA can be as high as 1000–1500 ml [23]. TKA blood loss can be divided into two components: visible blood loss and hidden blood loss [24, 25]. Visible blood loss consists mainly of intraoperative bleeding and postoperative drainage, whereas hidden blood loss refers to the accumulation of blood in the joint cavity and extravasation in the tissue spaces after major trauma or surgery, as well as haemoglobin loss due to haemolysis [26]. Tao Yuan et al. [27] suggested that visible bleeding after arthroplasty is associated with a hemolytic reaction following attack on red blood cells by a large number of oxygen radicals produced by stress. In our review of previous similar articles, we found that the findings of Li et al. [7] were similar to the present experiment, with significant differences in total and hidden blood loss between the two groups of patients. Dikmen et al. [4] also illustrated a difference in total blood loss between the empty tube and bone cement groups. The bone cement blocking of the femoral medullary canal in this experiment did reduce blood loss, probably because more gaps remained around the block after it was blocked. The bleeding from the femoral trophoblastic vessels in the medullary canal may enter the joint cavity or tissue space through these residual gaps. This stagnant blood in the joint cavity or tissue space may not only cause hidden blood loss, but also swelling and pain in the joint, which may affect the recovery of joint function.

There are also inconsistent results in terms of transfusion rates and drainage in previous articles, with Li et al. and Ko et al. finding lower transfusion rates in the bone cement group than in the empty tube group [6, 7], while other articles indicate no difference [3–5], which may be related to whether tranexamic acid was used intraoperatively. Tranexamic acid reduces bleeding and transfusion requirements in the perioperative period [28, 29].The transfusion rate in the study by Dikmen et al. [4] was partly due to a decrease in the use of tranexamic acid. In addition, there is a difference in the results of drainage flow between Dikmen İ et al. and Batmaz et al. [3, 5]. We compared them and found that the time of drainage tube removal was different, and since drainage flow increases with time, it is closely related to the time of removal and also to the use of perioperative anticoagulants [30, 31]. In this study we administered apixaban, a new oral anticoagulant that inhibits Xa factor activity, to patients after surgery. It is a new oral anticoagulant that inhibits Xa factor activity and has mild adverse effects due to its single target of action. Sayar et al. [32] found no myeloma chemotherapy patients had a thrombotic event as a result of receiving prophylactic apixaban. In our study there was no difference in transfusion rates or drainage between the two groups. We also took into account the comparison of postoperative DVT. We performed a lower limb ultrasound on the seventh postoperative day and found no difference in the incidence of DVT between the two groups, and the patients who had DVT had no symptoms associated with it at the time of follow-up, and it eventually resolved with anticoagulation.

This study has several drawbacks, firstly it is a single central retrospective study with a high potential for bias. Secondly the sample size was relatively small and the experimental data lacked convincingness; a large prospective study would have better validated our conclusions. Also, we only performed Doppler ultrasound on patients preoperatively and on the seventh postoperative day, which may have been missed due to the fact that Doppler ultrasound was only performed on patients with DVT-related symptoms only in the postoperative follow-up of patients after discharge from hospital. In addition, Doppler ultrasound is not the gold standard for the diagnosis of lower limb venous thrombosis and the likelihood of false negatives increases. When the Gross equation method is used to estimate blood loss, the true calculated total blood loss may be compromised due to the transfer of body fluids and haemolytic reaction to transfusion.

## Conclusion

In summary, sealing the femoral medullary canal with bone cement during TKA improves relative postoperative hypocoagulation and reduces postoperative blood loss, but has no effect on transfusion rates, drainage flow or the probability of developing DVT. Given that bone cement is relatively inexpensive and simple to obtain, it is possible to carry out these methods in the clinical setting.

## Data Availability

This published article includes all of the data created or analyzed during this investigation. We do not want to share our patients' information because it compromises their privacy.

## Abbreviations

TKA: Total knee arthroplasty
TEG: Thromboelastography
R: Reaction time
K: Clotting time
MA: Maximum amplitude
CI: Coagulation index
BMI: Body mass index
POD: Postoperative day
